# Patterns, facilitators and barriers to physical activity among Nigerian pregnant women

**DOI:** 10.11604/pamj.2022.42.321.31109

**Published:** 2022-08-30

**Authors:** Chidozie Emmanuel Mbada, Dolapo Adeola Ojo, Olabisi Aderonke Akinwande, Okechukwu Ernest Orji, Adebanjo Babalola Adeyemi, Kikelomo Aboyowa Mbada, Esther Kikelomo Afolabi

**Affiliations:** 1 Department of Medical Rehabilitation, College of Health Sciences, Obafemi Awolowo University, Ile-Ife, Nigeria,; 2 Department of Health Professions, Faculty of Health and Education, Manchester Metropolitan University, United Kingdom; 3 Department of Physiotherapy, University College Hospital, Ibadan, Nigeria,; 4 Department of Perinatology Obstetrics and Gynaecology, College of Health Sciences, Obafemi Awolowo University, Ile-Ife, Nigeria,; 5 Department of Political Science, Faculty of Social Sciences, Obafemi Awolowo University, Ile-Ife, Nigeria,; 6 Department of Nursing Science, College of Health Sciences, Obafemi Awolowo University, Ile-Ife, Nigeria

**Keywords:** Barriers, exercise, facilitator, Nigeria, physical activity

## Abstract

**Introduction:**

pregnancy is associated with sedentary behaviors and/or low levels of physical activity (PA). This study aimed to assess patterns, barriers, and facilitators of PA among pregnant women.

**Methods:**

a convergent parallel mixed method design study involving a concurrent collection of quantitative (n=198) and qualitative (n=36) data was carried out. Respondents were drawn from five selected health care facilities in Ile-Ife, Osun state, South-West, Nigeria. Physical activity was assessed using the pregnancy physical activity questionnaire. Focus group discussions were used to qualitatively explore barriers and facilitators of PA. Quantitative data were analyzed using descriptive and inferential statistics, while qualitative data were analyzed using thematic content analysis.

**Results:**

the mean total PA score for the population was 118.663±81.522 mets-min/wk. While it was 118.743±92.062 mets-min/wk, 113.861±72.854 mets-min/wk, and 25.429±87.766 mets-min/wk for the first, second, and third trimester respectively. The respondents engaged more in moderate (44.27±37.07) than vigorous (13.89±18.87) intensity PA. Respondents in the third trimester had the highest and the least scores for household-related PA (45.7±33.0) and vigorous-intensity PA (10.0±14.0) respectively. Major themes that emerged on enablers and barriers of PA engagement during pregnancy were related to intrapersonal, interpersonal, availability of specialized health personnel and policy for PA, good built environment/neighborhood factors, and pervading cultural beliefs and myths about pregnancy.

**Conclusion:**

moderate intensity and household-related PA were most common among Nigerian pregnant women. Contextual facilitators and barriers to PA during pregnancy were largely related to intrapersonal, interpersonal, environmental or organizational, policy, and cultural factors.

## Introduction

Pregnancy is a significant stage in a woman´s life [1] that is associated with considerable physiological and psychological changes that often predispose her to sedentary behaviors and/or low levels of physical activity (PA) [2]. Sedentariness or lack of PA during pregnancy, in turn, precipitates negative effects on the physical, psychological and psychosocial health of the woman [3,4]. On the other hand, being physically active during pregnancy is associated with a reduced risk of adverse pregnancy and birth outcomes; including preeclampsia, gestational diabetes, and preterm births [5]. Consequently, studies have recommended that women should initiate or continue exercise in most pregnancies [6,7] as it is safe for the mother and not harmful to the fetus [8,9]. While, only a few engage in exercises or sports activities during pregnancy, a substantial proportion of women stop exercising after they discover they are pregnant, thus leading to decrease levels of PA among them [10]. The causes of reduced PA in pregnancy are multifarious. Hormonal changes, [11] social, [12] physiological, [13] and psychological [12,14] factors have been implicated in many studies. In addition, factors not limited to beliefs and attitudes about PA [7,15] levels of knowledge and education, [16] phobia or safety concern of the pregnant woman and her physician, [17] race/ethnicity [18], and experience from previous involvement in PA [19] were reported to influence PA in pregnancy. Apart from certain factors that seem to be common as facilitators and barriers to PA in pregnancy, most of the factors that influence PA are context-specific [20]. However, the context-specific facilitators and barriers of PA among pregnant women seem to have been explored less in sub-Sahara Africa, compared with Western countries. Therefore, the objective of this study was to assess patterns, facilitators, and barriers to PA among Nigerian pregnant women.

## Methods

**Study design:** a convergent parallel mixed method design study involving a concurrent collection of quantitative and qualitative data was carried out.

**Study setting:** these respondents were recruited from the antenatal care clinics of five purposively selected facilities in Ile-Ife, Osun State, South-west, Nigeria. The facilities are: Obafemi Awolowo University Health Center, Obafemi Awolowo Teaching Hospital Complex, Osun State Primary Health Center, Oke-Ogbo, Primary Health center, Comprehensive Health Center, Enuwa and Urban and Comprehensive Health Center, Eleyele, Ile-Ife.

**Study population:** eligible respondents for the study were pregnant women who have had at least a second antenatal visit and were between 20 and 37 weeks of gestation, and who were between the ages of 18 and 35 years. Excluded from this study were pregnant women with a report of multiple gestations. The sample size for the quantitative aspect of the study was calculated based on a formula by Daniel [21]


$$n=\frac{{\left(Z\right)}^{2}\times p(1-p)}{d^{2}}$$

Where n= desired sample size, Z= 95% confidence level, it is 1.96, p= expected proportion in population and d= absolute error or precision. A sample size of 196 was calculated, however, 216 was estimated to allow for 10% non-response or invalid data. A total of 198 purposive respondents participated in the study. Also, a sample size of 30 is adjudged large enough for a qualitative study to allow the unfolding of a 'new and richly textured understanding´ of the phenomenon under study [22]. A total of 36 pregnant women responded in the qualitative phase of the study.

**Data collection:** the following questionnaires were used to collect data 1. Pregnancy physical activity questionnaire (PPAQ). The PPAQ developed by Chasan-Taber *et al*. [23] was used to quantitatively assess PA among pregnant women. The PPAQ consists of 33 items that aim at assessing different day-to-day activities (including household/ caregiving, occupational, sports/exercise, transportation, and inactivity) and how much time is spent doing each of the activities. To score the questionnaire, the total duration is multiplied by 7. The intensity was then calculated using field-based measurements and metabolic equivalent of task ((MET) values [23,24]. The PPAQ score is expressed in mets-minutes/week). Focus group discussion (FGD) interview guide. A FGD guide adapted from the Carolina Population Center´s PA and weight gain in pregnancy study [25] was used in this study. The FGD guide was subjected to face and content validity by experts in women´s health and PA studies at the Obafemi Awolowo University, Ile-Ife, Nigeria. In order to explore PA patterns, enablers, and barriers, the guide was adapted around the key issues in order to explore the depth of opinions on the subject. The moderator used the guide to elicit information that is relevant to the inquiry by asking questions and exploring answers as they arise. A full report of the discussion was obtained by note-taking and tape recording. A total of five FGDs were conducted, one in each of the facilities. Each FGD was composed of between 6 and 10 pregnant women. Table 1 shows the FGD guide used in the study. Furthermore, the FGD guide sought information on the practice and predictors of PA among women.

**Table 1:** pregnancy/physical activity study focus group guide

SN	Section	Detail
I	Introduction (10 minutes)	Introduction of the moderator
	Description of the programme	We are going to discuss your thoughts about physical activity behaviors before, during, and after pregnancy; you do not have to have any special knowledge to participate in this discussion; simply want to hear what you do in regards to physical activity behaviors particularly as it relates to pregnancy
	Setting the ground rules (standard)	
	Introduction of the participants	When you introduce yourself, please tell us your first name, what your due date is and the ages of any children you may already have
II	Warm up (5 minutes)	Desired outcome-we need to know what their definition of being physically active is
III	Prior to pregnancy (20 Minutes)	Desired outcomes insights into what were their physical activity behaviors prior to pregnancy first I want to talk to you about your physical health before you became pregnant
	What types of physical activities did you do before pregnancy? were you satisfied with your level of physical activity	Probe for activities both at leisure, at home, and at work If not, tell me more about why you were or were not satisfied with your PA
IV	During pregnancy (25 minutes) desired outcome insights into what has changed in regards to physical activity since they became pregnant	Next I would like you to think about your physical health choices during this pregnancy
	Tell me in what ways your physical activity or exercise at work and home changed since you became pregnant? what advice have you been given about participating in physical activity during pregnancy? what specific recommendations have you heard or been told about physical activity during pregnancy?	Has it increased or decreased? explain; probe reasons for the change: personal factors (e.g medical complications, time, money, health, stress, motivation), social factors (e.g. support of friends, family, husband), physical environmental factors (e.g. safety, where you live, access to facilities, weather),cultural and societal factors(e.g. beliefs, traditions, norms, etc.) Probes: who gave you or where did you get this information? was the advice clear/did you understand the advice? did you follow it or ignore it? explain list probes: from whom did you hear the information (family, partner, friends, and healthcare provider?) was the advice clear/ did you understand the advice? How important is it to you to follow the recommendations?
V	Barriers and facilitators to physical activity in pregnancy	What things do you consider as barriers or facilitators to effective physical activity in pregnancy? Is there any other thing you will want to tell me about physical activity in pregnancy?
VI	After the baby is born (15 minutes)	Desired outcome-insights into the importance of getting back to ideal weight and types of plans to achieve this goal; now, let's talk about your plans for physical activity participation after the baby is born
VII	Interventions (10 minutes)	
	We have been discussing things which influence your physical activity and your thoughts about inactivity during your pregnancy; now it's time for you to tell me what would make it easiest for you and for women like you to be active (probes: family/home;neighborhood, healthcare setting, work, other, again, let your imaginations run--is there anything else?)	
VIII	Closure (5 minutes)	
	After you leave today, what kinds of things will you tell your friends about being in today's discussion? If you have not filled out the two forms at the start of the focus group please, kindly help fill them	

Thank you for participating!!!

**Statistical analysis:** descriptive statistics of mean, standard deviation, and frequency distribution were used to summarize the sociodemographic variables of respondents. IBM SPSS (statistical package for social sciences) was used for statistical analysis. Verbatim transcription of the qualitative data collected from the focus group discussions was carried out. The interview transcripts were indexed and mapped according to recurring themes, and were analyzed using thematic content analysis. Physical activity was computed based on the PPAQ score. Average weekly energy expenditure (MET-h´week 1) was calculated by multiplying the duration of time spent in each activity by its intensity, following the computation guide described by Chasan-Taber *et al*. [23].

**Ethical consideration:** ethical approval for the study was obtained from the Health Research and Ethics Review Committee of the Institute of public health, Obafemi Awolowo University Ile-Ife, Nigeria (IPHOAU/12/945). All participants were informed of the purposes and procedures of the study and all provided both verbal and written consent.

## Results

**General characteristics:** a total of 198 and 36 respondents participated in the quantitative and qualitative aspects of the study. The socio-demographic, obstetrics, and clinical characteristics of the respondents are presented in Table 2. The mean age of respondents in this study was 29.04±3.74 years. The respondents were mostly civil/public servants (39.4%) and of the Christian religion (73.7%). About half of the respondents (48.0%) were in their second trimester and 88.9% still engaging in active work during their pregnancy. The majority of the respondents had no known pathological condition (91.4%) and were multiparous (45.5%).

**Table 2:** social-demographic, obstetrics and clinical characteristics of the respondents (N=198)

Variable		Frequency	Percentage	x̄SD
**Age**	<25yrs	43	21.7	29.04±3.74
	25-30yrs	87	43.9	
	31-35yrs	68	34.3	
**Marital status**	Married	188	94.9	
	Divorced	1	0.5	
	Single	9	4.5	
**Occupation**	Civil/public service	78	39.4	
	Trading/business	46	23.2	
	Studen	26	13.1	
	Artisans	37	18.7	
	Unemployed	11	5.6	
**Religion**	Christian	146	73.3	
	Islam	50	25.5	
	Others	2	1.0	
**Ethnicity**	Yoruba	157	79.3	
	Igbo	31	15.7	
	Hausa	9	4.5	
	Others	1	0.5	
**Trimester**	First	36	18.2	
	Second	95	48.0	
	Third	67	33.8	
**Parity**	Nulliparous	57	28.8	
	Primiparous	51	25.8	
	Multiparous	90	45.5	
**Work during pregnancy**	Yes	176	88.9	
	No	22	11.1	
**Hypertensive**	Yes	14	7.1	
	No	184	92.9	
**Diabetic**	Yes	1	0.5	
	No	197	99.5	
**Ulcer**	Yes	2	1.0	
	No	196	99.0	

**Quantitative data:** Table 3 shows the mean, percentile, and type of PA among the respondents (across the three trimesters). Mostly performed by the respondents were household PA (39.825±33.222) and sports (41.945±33.039) PA. The PA type with the highest and lowest mean score was moderate (44.27±37.07) and vigorous (13.89±18.87) intensity PA. The mean PA score was highest in the third trimester (125.4±87.8). While household-related PA was highest in the third trimester (45.7±33.0), the lowest mean PA score for vigorous-intensity PA (10.0±14.0) was also observed (Table 4).

**Table 3:** mean, percentile and physical activity types among pregnant women (for first, second, third trimesters, and combined data) N=198

Variable	Mean±SD	Median	Minimum	25^th^	50^th^	75^th^	95^th^	Maximum
First trimester (n=36)								
Sedentary (METs- mins/wk)	28.167± 28.016	15.450	0.430	9.172	15.450	37.350	97.680	97.680
Light intensity	38.213±35.887	22.310	1.750	15.007	22.310	51.983	123.026	125.160
Moderate intensity	34.922±22.633	35.887	3.150	16.500	35.710	52.843	78.692	85.390
Vigorous intensity	18.062±21.528	6.4 15	0.000	4.163	6.415	27.693	67.260	7.260
Household	38.174±40.511	23.630	1.550	9.813	23.630	51.370	104.762	111.460
Occupational	7.869±11.385	3.540	0.000	0.100	3.540	14.540	27.220	55.100
Sport	42.918±31.939	38.390	2.250	14.923	38.390	62.795	104.762	111.460
Total physical activity	118.743±92.062	92.750	25.040	53.650	92.750	151.040	349.085	351.550
**Second trimester (n=95)**								
Sedentary (METs- mins/wk)	23.736±19.359	15.450	0.230	10.200	5.610	36.000	63.882	85.870
Light intensity	33.410±27.339	23.310	2.010	15.600	23.300	47.690	86.026	135.040
Moderate intensity	42.365±37.703	35.710	0.250	14.670	33.750	60.560	112.066	250.030
Vigorous intensity	15.035±20.468	6.415	0.000	1.070	4.760	16.190	60.004	91.380
Household	36.293±29.989	27.830	0.6 20	12.280	27.830	47.440	100.03	126.260
Occupational	6.964±22.491	0.240	0.000	0.100	0.240	3.440	26.920	150.000
Sport	42.504±34.003	34.003	1.140	14.430	34.510	59.020	108.762	173.310
Total physical activity	113.861±72.854	96.680	18.940	56.770	96.680	166.470	275.096	370.250
**Third trimester (n=67)**								
Sedentary (METs- mins/wk)	27.118±29.286	17.680	0.000	9.100	17.680	41.77	83.786	138.490
Light intensity	34.844±22.039	29.286	2.490	20.180	29.230	47.050	83.352	115.090
Moderate intensity	51.998±±41.149	43.550	0. 670	21.280	43.550	75.150	137.280	184.220
Vigorous intensity	10.035±13.924	4.320	0.000	1.070	4.320	16.380	35.590	89.230
Household	45.7206±32.99	37.810	0.280	18.570	37.810	65.290	112.120	131.170
Occupational	10.473±21.624	2.500	0.0 00	0.140	2.500	14.400	58.160	119.040
Sport	40.631±32.678	34.980	0.380	14.210	4.980	54.690	111.522	162.430
Total physical activity	25.429±87.766	92.850	12.430	67.320	92.850	150.420	323.616	427.48
**Combined (n=198)**								
Sedentary (METs- mins/wk)	25.686±24.684	15.650	0.000	9.900	15.655	36.900	68.670	138.490
Light intensity	34.769±27.409	24.965	1.750	16.350	24.965	47.462	87.404	135.040
Moderate intensity	44.271±37.075	36.570	0.250	16.730	36.575	60.805	116.247	250.030
Vigorous intensity	13.893±18.869	5.010	0.000	1.070	5.010	16.600	58.962	91.380
Household	39.825±33.222	29.350	0.280	14.750	29.350	55.270	108.633	155.020
Occupational	8.316±20.580	0.240	0.000	0.100	0.240	5.940	44.140	150.000
Sport	41.945±33.039	35.460	0.380	14.430	35.460	57.240	107.658	173.310
Total physical activity	118.663±81.522	92.940	12.430	61.077	92.940	152.382	307.818	427.480

**Table 4:** frequency distribution of physical activity level of pregnant women based on demographic factors (N=198)

Variable		Physical activity (PA) level		
		Low	Moderate	High
		n (%)	n (%)	n (%)
**Age**	<25yrs	16	12	15
	25-30yrs	44	16	27
	31-35yrs	34	19	15
**Marital status**	Married	90	44	54
	Divorced	0	1	0
	Single	4	2	3
**Occupation**	Civil/public service	32	15	31
	Trading/business	24	13	9
	Student	15	4	7
	Artisans	16	12	9
	Unemployed	7	3	1
**Religion**	Christian	68	35	43
	Islam	25	11	14
	Others	1	1	0
**Ethnicity**	Yoruba	73	37	47
	Igbo	17	7	7
	Hausa	3	3	3
	Others	1	0	0
**Trimester**	First	16	10	10
	Second	46	23	26
	Third	32	14	21
**Parity**	0	34	10	13
	1	16	17	18
	2	25	11	18
	3	15	8	7
	4	4	1	1

### Qualitative data

**What is physical activity:** the concept of PA seems to be understood by a majority (73%) of pregnant women. *“To the best of my knowledge, it (PA) means doing our daily activities, you wake up in the morning, doing house chores, go to work,” (MKD)*. Although, some of these respondents misconstrue PA to be synonymous with exercise and physical fitness. *“Physical activity is normal daily works like house chores and work (occupation) and exercise” (SDG)*.In addition, respondents submit that taking care of children is essentially a major part of PA. *“at least we have to prepare for children in the morning is part of physical activity” (PKT)*. Respondents mostly attribute PA to household chores; involving cooking food, sweeping the floor, washing plates, bathing, and dressing children (61%). However, PA was rarely associated with leisure among women (Table 5).

**Table 5:** perceptions about physical activity, sources of information and perceived changes/factors in physical activity during pregnancy

Theme	Frequency	%
**What is physical activity? (n=36)**		
Correct response	26	73.0
Incorrect response	10	27.0
**Types of physical activity identified (n=36)**		
Leisure time	1	2.7
Work/occupation	8	22.0
Household	22	61.1
Active transportation	6	16.7
Outdoor	3	8.3
**What are the ways pregnant women can be physically active? (n=22 )**		
Leisure time	8	36.4
Work/occupation	8	36.4
Household	11	50.0
Active transportation	4	18.2
Outdoor	0	0
**Types of prenatal physical activity identified (n=36)**		
Leisure time	3	8.3
Work/occupation	13	36.1
Household	18	50.0
Active transportation	6	16.7
Outdoor	1	2.7
**Effect of pregnancy on level of PA**		
Increase	3	8.4
Decrease	30	83.3
Not changed	3	8.4
**Factors responsible for change in physical activity level**		
Personal (e.g time, money, health, stress, motivation, medical complications)	26	72.2
Social (e.g. support of friends, family, husbands)	1	2.7
Cultural and societal (e.g. beliefs, traditions, norms etc)	3	8.4
**Type of advice received on physical activity in pregnancy**		
Be active	9	20.5
Avoid stress	11	25.0
Maintain good posture	10	22.7
Eat good food (nutrition)	15	34.1
Respect myths about pregnancy	12	27.3
**Source of advice on physical activity in pregnancy**		
Family	13	29.5
Friends/neighbors	8	18.2
Health personnel	14	31.8
Traditions	8	18.2

**What are the ways women can be physically active:** the respondents submit that involvement in household chores will suffice for a pregnant woman. *“The things we (pregnant women) can do as part of PA include washing clothes, washing plates then if you have other kids in the house take care of them, move from one area to the other” (MKT)*. Nonetheless, some of the respondents, in addition to doing household chores submit that active working (occupation) and exercise are ways of improving PA in pregnancy. *“There are different types of exercise, walking for like 30 minutes down the street, maybe every day is good for the pregnant women also ” (SKT)*. Nonetheless, there were some misconceptions about how to improve PA among pregnant women. For example, some said *“eating energy giving food” (MKO), and “use your drugs appropriately and eat different fruits” (MKY)*.

**Types of prenatal physical activity:** most of the respondents revealed that they were involved in rigorous household activities involving lifting or carrying heavy loads, fetching water from the well water, and backing babies. *“Lifting of heavy loads, like a pail of water to flush by the time you´re gaining weight (because of pregnancy) you´ll discover you cannot carry many loads” (MKD)*. Other common outdoor activities reported include trekking long distances and occupational activity participation in exercises *“I work, to the extent, I can trek anywhere but now, we can work well before but now, we cannot work well again”(SDG)*. For some of the respondents, leisure time activity was more of going to religious programs, while few still will engage in leisure walking *“I was also active with church activities and all. I still do most of these things” (RSV)*.

**Perceived meaning and types of physical activity:** the types of PA identified were leisure-time PA, work/occupation, household, active transportation, and outdoor with household PA being the most mentioned type of PA. The perceptions about PA, sources of information, and perceived changes/factors in PA during pregnancy (Table 5).

**Changes and factors affecting physical activity since pregnancy:** the general consensus among the respondent was that PA was markedly reduced since being pregnant. *“My physical activity level has reduced definitely because the level of energy is lower. So everything is slower you know you are carrying a baby. It takes a lot from you” (MKD)*. Some of the women assert that there is strength sharing between the mother and fetus leading to fatigue and the attendant drop in PA. *“You know, another person is inside, for some it may be one or two (twin), part of the energy we have before is what the baby has taken” (RMD)*.

**Advice on participating in physical activity during pregnancy:** common advice given were on being active, avoiding or relieving stress, posture management, nutrition and respect for myths. On being active a respondent reported: *“It is recommended for us to sweep using standing broom if we cannot bend down, we are told other ways to do these things that will make it easier”(PRD)*. On avoiding stress, some of the respondents stated *“We heard the advice that we should not engage in strenuous work, that our legs must not be swollen more than necessary, they said we must not see blood on our body,” (SRT)*. On nutrition, some of the respondents submit *“my brother-in-law used to advise me not to eat plantain, a friend told me to drink pawpaw water because it prevents jaundice” (SMD). “My sister-in-law used to tell me not to take cold drinks, because it makes the baby´s head grow big ... I follow the advice once in a while. I still take a cold drink when I feel like it, especially when they are not at home” (MRD)*. Others forms of food to be avoided as reported by the women were bitter yam, snail, okra, and plantain. On posture and body ergonomics in pregnancy, the advice given was expressed thus: *“I am told not to bend down too much to sweep or wash clothes. I was told to sit down often so that my baby will not aspirate blood” (MDG). “They say bending down may cause blood to enter the baby´s eyes,” (NSD)*. The pregnant women in this study were advised also to adhere to certain cultural tenets, as revealed by some of the excerpts *“I have been advised not to go out in the afternoon sun around 1-3 pm, and that I should always attach a pin to my cloth before going to the market” (PRD).“We are advised to add a pin to our clothes before going out in order to scare evil spirits away. I don´t want anything to harm my child, so I adhere to this advice” (RPD). “They used to say if we mistakenly find ourselves outside around 1 pm we can take a small stone, put it in our ear and continue going, it was given by mothers before us, I follow them, they are good” (SDG)*. However, not all pregnant women believe in the cultural tenets. *I am a Muslim, I don´t have taboos but they used to say pregnant women should not walk in the sun around 1 o´clock. I don´t really follow them ”(SKD)*.

**Perceptions about post-partum engagement in physical activity:** various perceptions on post-delivery PA participation were expressed; *“When one gives birth, one should not be lazy, one should just do house chores and the stress of taking care of the baby is enough to make somebody to lose weight” (MRD). “After one delivers the weight sheds by itself, one does not have to do anything or use any drugs, it happened like that after my first baby” (NSB)*.

**Facilitators of physical activity:** some respondents believe that having good health is an important factor in PA engagement. *“I think good health is a facilitator, When you are healthy you will be able to do a lot of things” (MKY)*. Some believe that self or getting motivation by others to engage in PA is important (Table 6). *“Because I don´t have a choice, I am a working-class woman, when I realize that I do what I have to do” (PND).“My husband motivates me, if I am tired, he´ll wake me up, it´s time for this, time for that, so he always keeps me going”(PGS)*. Having money and availability of good food were also reported as facilitators of PA in pregnancy. *“Money gingers somebody to be very active, when things are going well with the family one is happy and doing everything is easy” (MSB)*. Availability of good built environment or neighborhood was another facilitator of PA in pregnancy. *“Good roads is necessary, almost all the roads are bad and is not suitable for walking for a pregnant woman” (MKY)*. Availability of specialized health personnel and policy for PA in pregnancy. *“Workplace should be mandated to give a day off for antenatal where physical activity program will be carried out without having to rush back to work”(RSV)*.

**Table 6:** facilitators and barriers of physical activity in pregnant women

Level	Descriptive themes	Frequency	Percentage
**Facilitators in pregnant women**			
Intrapersonal	Health	11	25.0
	Time consciousness	1	2.3
	Self determination	3	6.8
	Motivation	2	4.5
Interpersonal	Social support (family, friends etc)	13	29.5
	Financial stability	6	13.6
Availability of specialized health personnel and policy for physical activity		5	11.4
Good built environment/neighborhood		3	6.8
**Facilitators and barriers of physical activity in pregnant women**			
Intrapersonal	Pregnancy-related symptoms and limitations	8	33.3
	Time constraints	1	4.2
	Perceptions of already active	1	4.2
	Lack of motivation	5	20.8
	Mother-child safety concerns	1	4.2
Interpersonal	Lack of advice and information	3	12.5
	Lack of social support	2	8.3

**Barriers to physical activity in pregnancy:** ill health or co-morbid conditions in pregnancy and financial challenges were considered as barriers; *“health challenges and weakness affects one from being physically active” (MKY). “Money is a stumbling block because if there is no money to take care of oneself, eat good food, one may not be able to do anything, because the body will be weak”(MYB)*. Personal factors such as pregnancy-related physiological changes, mood and depression, and having wrong advisers were implicated as barriers (Table 6); *“when there is nobody to exercise with, like walking now, it is boring to walk” (SRT).“When you are told that exercising or stressing the body can result in miscarriages or other complications, you will be careful”(SRT). “Some people are still fighting with their past and it will affect their present, causing depression which will render them physically inactive” (PDG)*. Some cultural beliefs is reported as an impediment to PA in pregnancy *“our culture and what our elders tell us is one thing that can be a barrier, for example in some families they say their pregnant women must not work least they have a miscarriage so the woman is not allowed to work until she give birth” (MKT)*.

## Discussion

The PA scores obtained in this study varied according to pregnancy trimester. Women in the third trimester had the highest PA score. Contrary to this finding, previous studies found high levels of PA during the second trimester of pregnancy [26,27]. However, the finding of this study may be attributed to anecdotal evidence in the study setting where women in the advanced stage of pregnancy usually want to be physically active, as it is believed to aid, especially, spontaneous vagina delivery process, owing to palpable aversion for cesarean section [28]. A study in the context where this study was conducted found that cesarean section among the Yoruba of Western Nigeria is treated with suspicion, aversion, misconceptions, fear, guilt, misery, and anger [29]. Anecdotally, full-term women in the study context are often encouraged to engage in more than usual activities such as rigorous walking, pounding yam to make iyan (a staple yam meal in the study location), or making cassava flour meals, which are laborious household chores. Based on PA types, pregnant women in the third trimester had the highest overall mean in household-related PA. This finding is supported by the report of Florindo *et al*. [30] who reported that the highest of all reviewed PA types among pregnant women was household intensity PA. Adeniyi *et al*. [31] explain that the third trimester is a period where pregnant women, usually embark on maternity leave which may make them spend more time at home than at work. From this study, vigorous intensity and occupational PA were comparable across trimesters. A possible reason may be that many women once they discovered they are pregnant, reduce engaging in occupational activities and may feel more comfortable and safer doing household activities irrespective of trimesters [32].

From this study, pregnant women in the third trimester have the least mean score for vigorous-intensity PA. This finding is generally consistent with previous research indicating a significant decline in time spent in total and vigorous leisure PA and stable levels of moderate leisure PA from pre-pregnancy to pregnancy [33,34]. It was also observed that there was no significant association between PA levels and the sociodemographics of pregnant women. Findings on the association between PA and sociodemographics of pregnant women have been somewhat inconsistent [35,36]. In the qualitative results in this study, a large proportion of the women seem to exhibit some knowledge of PA, however, the knowledge was generally within the context of household PA. Kader and Naim-Shuchana [37] in a review reported that there is limited knowledge, even about participation in elite sports in pregnancy even though exercise during pregnancy does not increase any risk of adverse pregnancy or birth outcomes, not even for elite athlete women. Nigerian pregnant women in this study, have limited understanding of the different domains of PA, other than engagement in household chores. Some of the women, in addition to engaging in household chores, submit that active working (occupation) and exercise are ways of improving PA in pregnancy. Most of the women in this study stated that they were involved in rigorous household activities involving lifting or carrying heavy loads, fetching water from the well, etc. This finding buttress the quantitative result that household PA is the most common type of PA among Nigerian pregnant women. This is probably due to norms of the ethnic groups in Nigeria that make household chores mandatory for the female gender. This finding support previous reports about West Africa that women usually work longer hours a day than males in similar circumstances. Specifically, that women's working week is longer than 64 hours, whereas for men it is only about 32 hours. About half of women's time is spent on domestic tasks, but even then women spend more time on agriculture than men do (26 hours/week compared with only 12 hours/week for men) [38]. Sumra and Schillaci [39] submit that multiple role engagement in women, even at a relatively high level as experienced by “superwomen”, is not associated with significantly higher stress, or reduced life satisfaction.

Most of the women in this study acknowledged that their PA became markedly reduced since being pregnant, which is in line with previous reports [40]. Some of the women rehearsed fatigue as a major factor influencing change in PA. Other studies have noted the impact of fatigue on women´s level of PA [9,41]. Advice/information, myths, and misconceptions about PA were mostly gotten from family and friends. This study´s finding is in agreement with a previous study by Prochaska *et al*. [42]. From a practical perspective, the summary of factors that facilitated women´s engagement in PA during pregnancy includes social support; personal factors (such as motivation, self-determination, and time consciousness); availability of money and good food; availability of a good built environment or neighborhood, as well as, availability of specialized health personnel and policy for PA participation in pregnancy. Some of these factors have been identified by other studies [43,44]. Women in this study identified a number of factors that hindered their capacity to be physically active. Consistent with reports from other studies, many of these were health or practical issues, including pregnancy-related symptoms like feeling tired or weak and decreased motivation and time, which are factors that are often associated with working and family commitments. Some barriers are transient, but some were present throughout the entire period of pregnancy. Perceived barriers arose from women´s concern about the risks of being physically active for either themselves or their fetus. Other barriers include; time constraints; perception of already being active, dearth of money; lack of motivation and company; wrong advice; mood, and depression. Some of these are supported by the literature [33,45,46]. A systematic review of a qualitative and quantitative approache to perceived barriers to leisure-time physical activity during pregnancy concludes that mother-child safety concerns, lack of advice/information, and lack of social support were also importantly emphasized in pregnancy-related barriers to be targeted in future interventions [46]. The responding pregnant women in this study based on sociodemographic characteristics were relatively young and were only recruited from selected hospitals from only one region in Nigeria. Therefore, the findings of this study can only be generalized to similar contexts, as an extrapolation of findings to developed settings may be challenging.

**Clinical implications:** there is a need for programmatic actions aimed at promoting PA among pregnant women by dispelling myths and eliminating barriers that limit participation in PA. Therefore, education and training on PA should be part of the continuum of care at antenatal care setting.

## Conclusion

Moderate intensity household physical activity is preponderant among Nigerian pregnant women, especially in the third trimester of pregnancy. Major themes that emerged on enablers of PA engagement during pregnancy were related to intrapersonal (absence of pregnancy-related symptoms, time availability and self-determination), interpersonal (availability of social support and financial stability), availability of specialized health personnel and policy for PA, and good built environment/neighborhood factors. While the barriers were mostly related to intrapersonal (having a sense of impaired health requiring PA engagement, time constraints, lack of motivation, perception on usefulness of PA and safety concerns), and interpersonal (lack of advice/information and social support) factors, and pervading cultural beliefs and myths about pregnancy.

### What is known about this topic

- Physical activity participation in pregnancy is associated with better outcomes for mother and fetus, including controlled weight and reduced predilection for obesity;
- Physical activity is generally considered safe for both the pregnant woman and the fetus;
- Women face significant barriers to participating in require physical activity needed for the healthy pregnancy.

### What this study adds

- Pregnant women, especially those in the third trimester engage in moderate intensity household physical activity;
- Absence of pregnancy-related symptoms, personal characteristics, financial stability, availability of social support and physical built environment are facilitators of physical activity in pregnancy;
- Presence of health challenge during pregnancy, physical and psychosocial factors, safety concerns and pervading cultural beliefs and myths about pregnancy prevent pregnant women from participating in physical activity.
